# Positive epistasis between viral polymerase and the 3′ untranslated region of its genome reveals the epidemiologic fitness of dengue virus

**DOI:** 10.1073/pnas.1919287117

**Published:** 2020-05-04

**Authors:** Ayesa Syenina, Dhanasekaran Vijaykrishna, Esther Shuyi Gan, Hwee Cheng Tan, Milly M. Choy, Tanamas Siriphanitchakorn, Colin Cheng, Subhash G. Vasudevan, Eng Eong Ooi

**Affiliations:** ^a^Program in Emerging Infectious Diseases, Duke-National University of Singapore Medical School, 169857 Singapore;; ^b^Saw Swee Hock School of Public Health, National University of Singapore, 117549 Singapore;; ^c^Department of Microbiology, Biomedicine Discovery Institute, Monash University, Clayton, VIC 3800, Australia;; ^d^Department of Biological Sciences, National University of Singapore, 117558 Singapore;; ^e^Department of Microbiology and Immunology, Yong Loo Lin School of Medicine, National University of Singapore, 119228 Singapore;; ^f^SingHealth Duke-National University of Singapore Global Health Institute, 169857 Singapore

**Keywords:** dengue, epidemiologic fitness, sfRNA packaging

## Abstract

Dengue virus (DENV) is a global health threat, causing repeated epidemics throughout the tropical world. While low herd immunity levels to any one of the four antigenic types of DENV predispose populations to outbreaks, viral genetic determinants that confer greater fitness for epidemic spread is an important but poorly understood contributor of dengue outbreaks. Here we report that positive epistasis between the coding and noncoding regions of the viral genome combined to elicit an epidemiologic fitness phenotype associated with the 1994 DENV2 outbreak in Puerto Rico. We found that five amino acid substitutions in the NS5 protein reduced viral genomic RNA (gRNA) replication rate to achieve a more favorable and relatively more abundant subgenomic flavivirus RNA (sfRNA), a byproduct of host 5′-3′ exoribonuclease activity. The resulting increase in sfRNA relative to gRNA levels not only inhibited type I interferon (IFN) expression in infected cells through a previously described mechanism, but also enabled sfRNA to compete with gRNA for packaging into infectious particles. We suggest that delivery of sfRNA to new susceptible cells to inhibit type I IFN induction before gRNA replication and without the need for further de novo sfRNA synthesis could form a “preemptive strike” strategy against DENV.

## Significance

Dengue affects approximately one-half of the world’s population. While it causes regular and cyclical outbreaks throughout the tropical world, the molecular mechanisms that determine its epidemic potential remain poorly defined. The present work provides insight into the molecular determinants of epidemiologic fitness, which is critical for surveillance to identify dengue virus strains with a potential to cause outbreaks. This will allow for better implementation of control measures to intercept impending outbreaks before many lives are affected.

Dengue is the most common vector-borne viral disease globally. Dengue virus (DENV), which exists as four antigenically distinct serotypes, afflicts an estimated 100 million people each year with acute disease, some of which are life-threatening (1). The geographic footprints of DENV and its mosquito vector, *Aedes aegypti*, are both set to grow in the coming years with global warming and transboundary trade and travel (2). Without a vaccine that effectively prevents infection by all four DENV serotypes or a licensed drug that can rapidly knock down viral burden in dengue patients and thereby reduce the likelihood of DENV transmission, cyclical dengue epidemics will continue to plague global health.

Dengue epidemics are mostly thought to result from the emergence of a new serotype of DENV in populations with low serotype-specific herd immunity (3). However, several outbreaks have occurred in areas where new DENV strains emerged and displaced endemic strains without a change in either viral serotype or genotype. Such instances have been observed in Sri Lanka (4), Puerto Rico (5), Singapore (6), Vietnam (7), Nicaragua (8), Taiwan (9), and Brazil (10). In all of these outbreaks, phylogenetic analyses grouped the newly emerged DENVs into clades distinct from DENVs that circulated before the outbreak. These findings thus suggest the existence of genetic determinants of epidemiologic fitness. Defining the molecular underpinnings of such epidemiologic fitness of DENV not only improves our understanding of dengue virology, but also could be developed into DENV genomic surveillance that scales disease prevention measures based on genetically defined risk of outbreaks (11).

To gain insight into genetic determinants of epidemiologic fitness, we have taken advantage of the 1994 dengue outbreak in Puerto Rico. In this outbreak, sequence data of the DENV genome revealed the emergence of a clade of DENV2 (PR2B clade) in 1994 that displaced another DENV2 clade (PR1) that had circulated endemically before the outbreak (5). We previously found that the increased epidemiologic fitness of PR2B relative to PR1 was conferred in part by three mutations in the 3′ untranslated region (UTR) of the viral genome (12). These mutations led to production of more subgenomic flavivirus RNA (sfRNA) relative to genomic viral RNA (gRNA) in PR2B viruses (12). The formation of sfRNA is mediated by host 5′-3′ exoribonuclease (XRN1), which digests gRNA in a 5′ to 3′ direction but stalls when the enzyme encounters highly conserved secondary RNA structures in the 3′ UTR (13); thus, sfRNAs are 3′ UTR remnants (14, 15). These remnants are functional, however, as PR2B sfRNA binds the E3 ligase, TRIM25, in a sequence-dependent manner to reduce its polyubiquitylation activity on RIG-I, which is necessary for sustained and amplified type I IFN induction. Thus, a reduced IFN response could have enabled PR2B DENV2 to achieve higher viremia titers during human infection, increasing the likelihood of a symptomatic outcome as well as transmission to blood-feeding *Aedes* mosquito vectors (12), collectively increasing the incidence of dengue.

However, our focus on the 3′ UTR is driven primarily by curiosity as to how nucleotide substitutions in the 3′ UTR could impact the epidemiologic fitness of DENV. There were other substitutions in the coding regions of the genome that segregated PR2B from PR1, which also could have contributed to the increased epidemiologic fitness of PR2B viruses. To understand the chain of molecular events that led to the 1994 dengue outbreak, we reconstructed the viral ancestors and estimated the time in which the genetic variants emerged. We found that the 3′ UTR mutations were acquired as early as 1985, 9 y before the outbreak. The mutations that immediately preceded the 1994 outbreak were found mostly in the nonstructural 5 (NS5) gene, which encodes viral methyltransferase and RNA-dependent RNA polymerase (RdRP). We show here that these NS5 substitutions reduced gRNA abundance, and that a reduced gRNA replication rate was necessary for more favorable sfRNA formation. Unexpectedly, we found that increased abundance of sfRNA relative to gRNA further enabled sfRNA to be packaged into DENV envelope (E) protein containing infectious particles. Thus, on infection, sfRNA could be delivered to new susceptible host cells without further de novo synthesis. Our findings suggest that NS5 substitutions in PR2B viruses are necessary to reveal the immune evasive function of sfRNA.

## Results

### PR2B NS5 Substitutions Were Acquired Immediately before the 1994 Outbreak.

To systemically define the amino acid substitutions that could have contributed to the emergence of PR2B in 1994, we conducted ancestral state reconstruction on the codon phylogenies of all DENV2s isolated from Puerto Rico dating as far back as 1981. This was done by first estimating the tree topology using the maximum likelihood (ML) method using the general time-reversible (GTR) nucleotide substitution model in Randomized Axelerated Maximum Likelihood (RaXML v. 8) (16) and subsequently estimating genetic distances and ancestral sequences using phylogenetic analysis by maximum likelihood (PAML), version 4 (17). Whole-genome sequences were obtained from an online database, the National Institute of Allergy and Infectious Diseases (NIAID) Virus Pathogen Database and Analysis Resource (ViPR) (18). Mapping the mutations acquired by PR2B on to the branches of the tree revealed eight unique substitutions that led to the divergence of PR2B from PR1 (*SI Appendix*, Fig. S1). One of these substitutions was in NS1 (T164S), two were in NS3 (L31F and K418R), and five were in NS5 (S269N, K375R, R514K, R514K, and R891K) (*SI Appendix*, Fig. S1). In particular, three of the five amino acid substitutions that immediately preceded the expansion of the number of PR2B isolates were all in the NS5 protein. Thus, we focused our study on NS5.

**Fig. 1.**
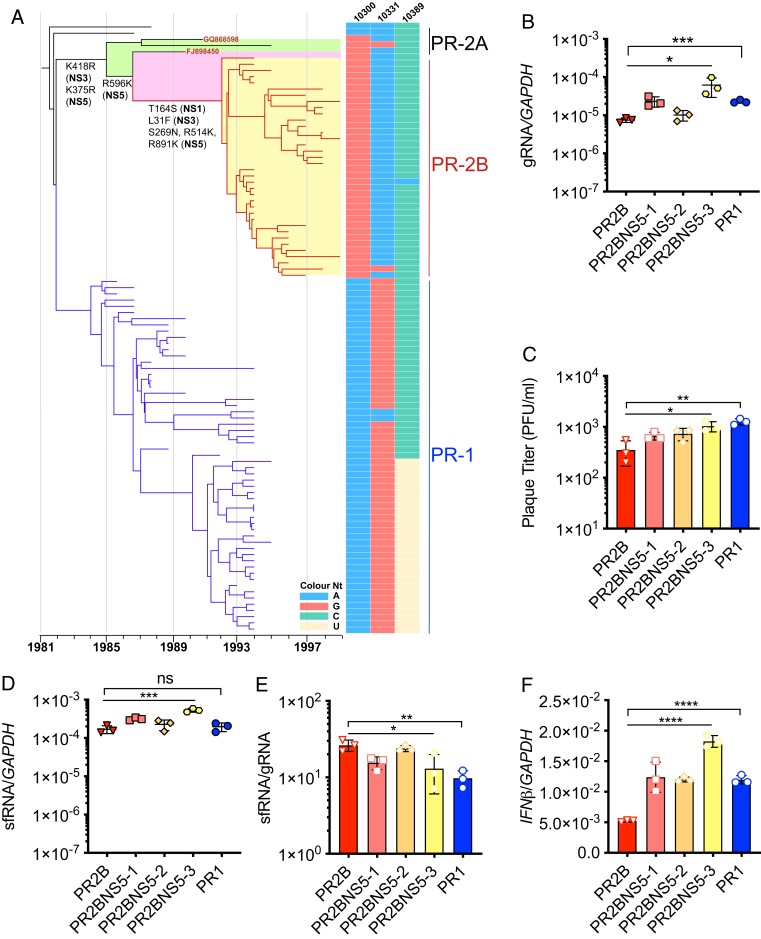
NS5 mutations in PR2B proceeded 3′ UTR mutations. (*A*) Temporally structured phylogenetic tree based on codon genome of DENV2 circulating in Puerto Rico between 1944 and 2010. Amino acid substitutions separating PR2A and PR2B are K418R (NS3) and K375R (NS5) in 1985 (95% CI, 1983.88 to 1986.88); R596K (NS5) in 1987 (95% CI, 1985.31 to 1988.24); and T164S (NS1), L31F (NS3), and S269N, R514K, and R891K (NS5) in 1992 (95% CI, 1991.48 to 1992.74). Adjacent to each tip are the three 3′ UTR nucleotide sequences at positions 10300, 10331, and 10389 that differ between PR1 and PR2B. GQ868598 and FJ898450 are PR2A viruses with identical 3′ UTR mutations as PR2B. (*B*) Quantification of gRNA levels at 24 hpi in Huh7 cells using qPCR. (*C*) Plaque-forming units determined from cell supernatants. (*D*) Quantification of sfRNA levels at 24 hpi in Huh7 cells using qPCR. (*E*) sfRNA:gRNA ratios quantified from intracellular gRNA and sfRNA levels. (*F*) Quantification of mRNA expression of *IFN-β* at 24 hpi using qPCR. Data are presented as mean ± SD. *P < 0.05; **P < 0.01; ***P < 0.001; ****P < 0.0001, unpaired *t* test or one-way ANOVA. ns, not significant.

Four of the five NS5 mutations differentiated PR2B from a sister PR2A clade containing DENV2s that were detected at low frequency (5). These mutations immediately preceded the expansion in the number of PR2B isolates (Fig. 1*A* and *SI Appendix*, Fig. S1). Sequentially, following the K375R substitution that occurred at the root of the PR2A and PR2B clades, R596K was followed by S269N, R514K, and R891K in the PR2B clade (Fig. 1*A* and *SI Appendix*, Fig. S1). We next ascertained when these NS5 mutations were acquired relative to the three nucleotide mutations in the 3′ UTR using Bayesian Evolutionary Analysis by Sampling Trees (BEAST) (19). The resulting maximum clade credibility (MCC) tree was juxtaposed against the 3′ UTR sequence at positions 10300, 10331, and 10389 (12). Interestingly, PR2A DENV2s isolated in 1990 and 1991 also possessed the same 3′ UTR mutations as PR2B (Fig. 1*A*), the common ancestor of which could be traced back to a node in 1985 (95% CI, 1983.88 to 1986.88). These findings suggest that the phenotype conferred by the PR2B 3′ UTR could not be manifested without additional mutations in the NS5 gene.

### NS5 Mutations Regulate gRNA Replication and sfRNA Production.

To elucidate the effect that NS5 mutations had on PR2B infection, we generated infectious clone of PR2B and used reverse genetics to rescue viruses that represented the nodes of the phylogenetic tree (Fig. 1*A*) as DENV2 evolved from PR1 to PR2B (Table 1). Inoculating these intermediates onto human hepatoma (Huh-7) cells at a multiplicity of infection (MOI) of 0.1 produced gRNA levels (Fig. 1*B*) and infectious viral progenies (Fig. 1*C*) that were intermediate to those of the control PR1 and PR2B viruses at 24 h postinfection (hpi). Levels of gRNA and plaque-forming units (pfu) of PR2B increased as NS5 substitutions in the PR2B genomic backbone was reversed to the PR1 residue. A statistically significant difference in sfRNA levels was observed, although the magnitude of change was small (Fig. 1*D*). Thus, with increased gRNA levels as PR1 NS5 sequences replaced PR2B NS5, the sfRNA:gRNA ratio was reduced (Fig. 1*E*). Correspondingly, *IFN-β* expression was increased (Fig. 1*F*).

**Table 1.** Infectious clones generated on PR1 and PR2B backbones bearing NS5 and/or 3′ UTR mutations by amino acid/nucleotide position

Clone	NS5					3′ UTR		
	269	375	514	596	891	10300	10331	10389
PR2B backbone								
PR2B	N	R	K	K	K	G	A	C
PR2BNS5-1	S	R	R	K	R	G	A	C
PR2BNS5-2	S	R	R	R	R	G	A	C
PR2BNS5-3	S	K	R	R	R	G	A	C
PR1 backbone								
PR1	S	K	R	R	R	A	G	T
PR13UTR	S	K	R	R	R	G	A	C
PR1NS5	N	R	K	K	K	A	G	T
PR1NS53UTR	N	R	K	K	K	G	A	C
PR1NS5-269	N	K	R	R	R	A	G	T
PR1NS5-375	S	R	R	R	R	A	G	T
PR1NS5-514	S	K	K	R	R	A	G	T
PR1NS5-596	S	K	R	K	R	A	G	T
PR1NS5-891	S	K	R	R	K	A	G	T

**Fig. 2.**
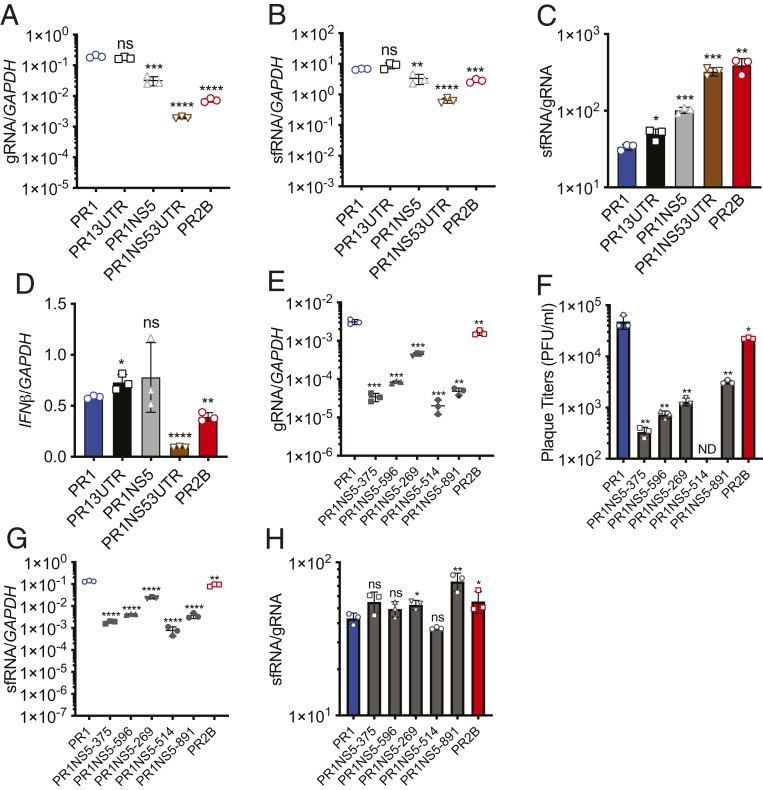
NS5 mutations result in retardation of virus gRNA and augments the sfRNA:gRNA ratio. (*A*–*D*) Quantification of gRNA levels (*A*), sfRNA levels (*B*), sfRNA:gRNA ratios (*C*), and mRNA expression of *IFN-β* (*D*) in primary monocytes by RT-qPCR. (*E*) Quantification of gRNA levels in infected A545 cells using qPCR. (*F*) Plaque-forming units determined from supernatant of infected cells. (*G*) Quantification of sfRNA levels in infected A545 cells using qPCR. (*H*) sfRNA:gRNA ratios quantified from gRNA and sfRNA levels. Data are presented as mean ± SD. *P < 0.05; **P < 0.01; ***P < 0.001; ***P < 0.0001, unpaired *t* test. ns, not significant.

To confirm the foregoing findings, we generated infectious clones with a PR1 genomic backbone and replaced its NS5, 3′ UTR, or both with those from PR2B (Table 1). Replacing PR1 NS5 with PR2B NS5, with (PR1NS53UTR) or without (PR1NS5) PR2B 3′ UTR, resulted in reduced gRNA levels in primary monocytes (Fig. 2*A*). In contrast, replacement of PR1 3′ UTR with that from PR2B alone (PR13UTR) did not result in a significant change in gRNA levels (Fig. 2*A*). Correspondingly, a greater increase in sfRNA:gRNA ratio was observed in PR1NS5-infected cells and PR1NS53UTR-infected cells than in PR13UTR-infected cells (Fig. 2*C*). *IFN-β* expression was likewise reduced in primary monocytes infected with PR1NS53UTR and PR2B DENV2, but not in cells infected with the other two mutants (Fig. 2*D*). That PR1NS5 failed to inhibit IFN-β induction (Fig. 2*D*) despite its sfRNA:gRNA ratio than PR1 is consistent with our previously suggested notion that sfRNA bound and inhibited TRIM25 in a sequence-dependent manner (12). These findings suggest that DENV NS5 and 3′ UTR interact under epistasis to reveal the function of DENV sfRNA.

Our findings in primary monocytes were reproduced in two different cell lines, the lung epithelial A549 (*SI Appendix*, Fig. S2 *A*–*D*) and Huh7 (*SI Appendix*, Fig. S2 *E*–*H*). Likewise, our qPCR approach to measuring the relative proportion of sfRNA to gRNA was validated using Northern blot analysis (*SI Appendix*, Fig. S2*I*). Furthermore, assessment of viral kinetics of all our mutant viruses in A549 cells was consistent with previous observations (12) in which PR2B gRNA levels were higher than PR1 gRNA levels at later timepoints (48 and 72 hpi) (*SI Appendix*, Fig. S2*J*). This phenotype was also found in PR1NS53UTR, but not in PR1NS5 or PR13UTR, further suggesting that epistatic interaction between DENV NS5 and 3′ UTR elicited the PR2B phenotype. We also examined the ability of our mutant viruses to infect *A. aegypti*, the natural vector of DENV. All viruses were viable and replicated well in these mosquitos (*SI Appendix*, Fig. S3).

We next determined whether each of these five amino acid substitutions in the NS5 protein play functional roles in reducing the rate of gRNA replication and increasing sfRNA relative to gRNA levels. We generated five infectious clones of PR1, each bearing one of the five PR2B amino acid residues (Table 1). All mutant viruses displayed significant reductions in gRNA (Fig. 2*E*) and pfu (Fig. 2*F*). Likewise, sfRNA levels were universally lower for all mutants (Fig. 2*G*). The sfRNA:gRNA ratio (Fig. 2*H*) increased for all but one of the mutants, although statistical significance was reached only in viruses with NS5 mutation at positions S269N and R891K. Of these, S269N was in the linker region of NS5, while the remaining four mutations were in the RdRp, three of which were arginine-to-lysine substitutions (R596K, R514K, and R891K). The arginine patch in the RdRp was previously found to be important for gRNA binding (20). Consequently, R-to-K substitutions could have resulted in reduced PR2B gRNA binding and thus viral replication (Fig. 2 *E* and *F*), which is consistent with our observation in all mutants using either the PR1 or PR2B backbone, where the reduction in gRNA appears to be the major determinant of the sfRNA:gRNA ratio. Finally, amino acid 891 was previously shown to be a part of the nuclear localization signal of NS5 translocation (21), although that study substituted the R amino acid with alanine; R891K did not appear to alter the nuclear localization of PR2B compared with PR1 NS5 protein (*SI Appendix*, Fig. S2*M*).

### Reduced gRNA Replication Is Required for Augmenting sfRNA:gRNA Ratios.

**Fig. 3.**
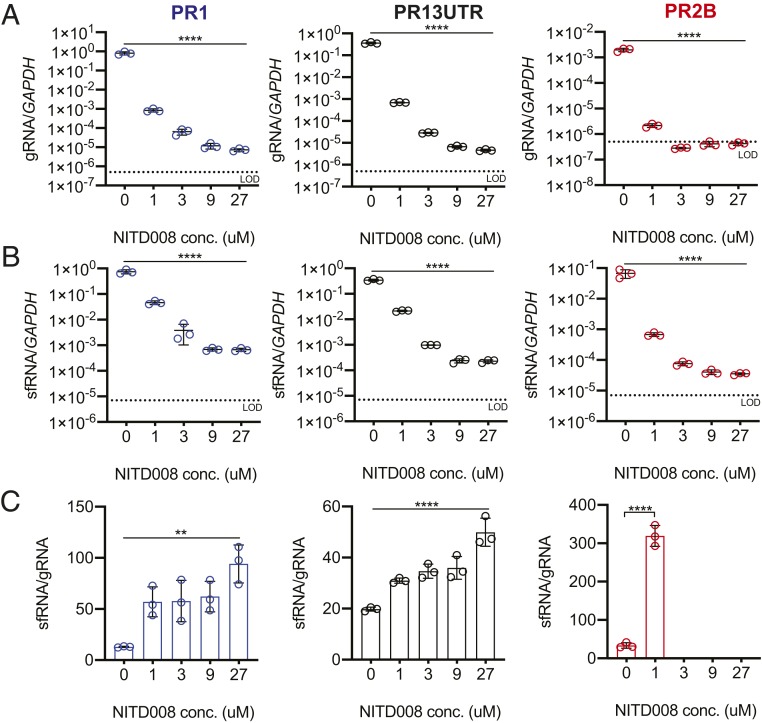
Reduced gRNA replication is required for an increased sfRNA:gRNA ratio. Quantification of gRNA levels (*A*), sfRNA levels (*B*), and sfRNA:gRNA ratios (*C*) in A549 cells with cotreatment of NITD008 at increasing concentrations. Ratios of sfRNA:gRNA were not calculated when the gRNA levels were at or below the limit of detection (LOD). Data are presented as mean ± SD. **P < 0.01; ****P < 0.00001, one-way ANOVA.

Reduced gRNA levels in PR2B compared with PR1 may be fundamental for the observed elevated sfRNA:gRNA ratio that forms the previously postulated “one-two punch.” Theoretically, given a fixed level of XRN1 expression, less gRNA substrate would result in an excess enzyme-to-substrate ratio for more complete digestion of uncapped gRNA to form sfRNA. If this explanation contributed to the observed increase in the sfRNA:gRNA ratio in PR2B viruses, then we should be able to increase the sfRNA:gRNA ratio in PR1 and PR13UTR by reducing gRNA replication. To test this notion, we repeated our experiment but with the addition of the antiviral compound NITD008, to inhibit DENV NS5 polymerase activity (22). To avoid completely eliminating DENV infection, we used NITD008 concentrations below those previously shown to fully inhibit DENV polymerase activity (22). Indeed, cotreatment of NITD008 yielded lower gRNA levels at 24 hpi for both PR1 and PR13UTR in a dose-dependent manner (Fig. 3*A*). Likewise, sfRNA levels were also decreased, but the reduction was not as great as that in gRNA levels (Fig. 3*B*). Correspondingly, the sfRNA:gRNA ratio in both PR1- and PR13UTR-infected cells increased with increasing doses of NITD008 (Fig. 3*C*). As a control, we also treated PR2B-infected cells with NITD008. Similar to the PR1 and PR13UTR, the sfRNA:gRNA ratio was increased with increasing concentration of NITD008 (Fig. 3*C*); however, gRNA levels dropped below the limit of detection with 3 μM of NITD008, unlike those for PR1 and PR13UTR (Fig. 3*A*). This finding supports our suggestion that the amino acid substitutions in NS5 reduced the efficiency of gRNA binding and replication. Taken together, our findings suggest that the PR2B NS5 substitution reduced gRNA levels for more favorable sfRNA production relative to gRNA.

### High sfRNA:gRNA Ratios Enable Encapsidation of sfRNA in Envelope-Containing Infectious Particles.

**Fig. 4.**
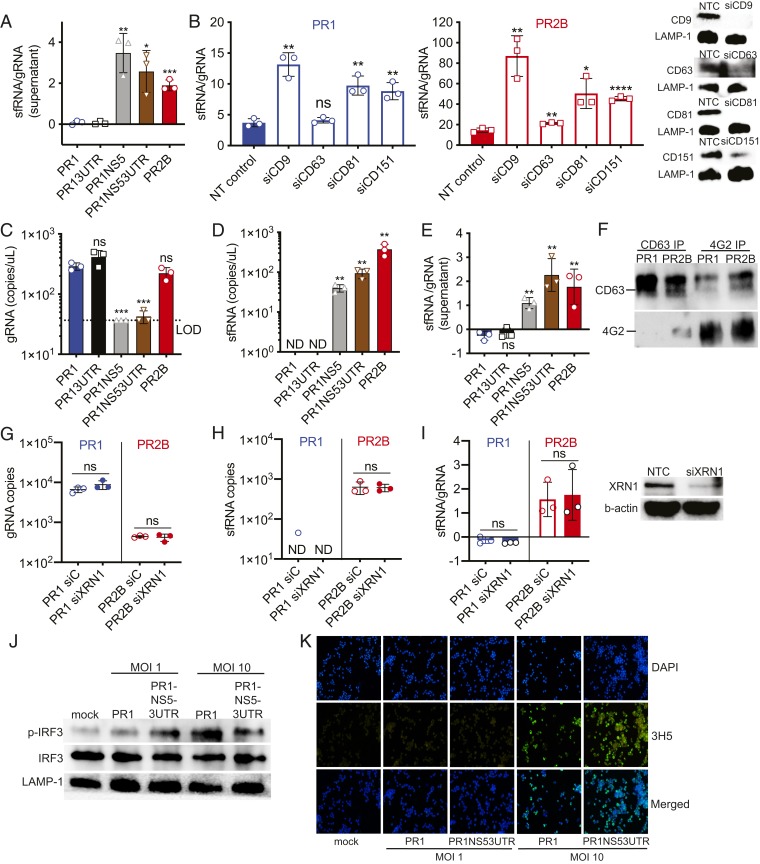
PR2B sfRNA is packaged inside E protein-containing infectious particles and transmitted into new susceptible cells. (*A*) Quantification of sfRNA:gRNA ratios in supernatant of A549 cells at 24 hpi by qPCR. (*B*) Quantification of gRNA:sfRNA ratios in supernatant of A549 cells on knockdown of CD9, CD63, CD81, and CD151. NTC served as a reference point for assessment of significance. (*C*–*E*) Quantification of gRNA copy numbers (*C*), sfRNA copy numbers (*D*), and sfRNA:gRNA ratios (*E*) from 4G2 pull-down precipitate. LOD, limit of detection; ND, no detection. PR1 served as a reference point for assessment of significance. (*F*) Western blots of CD63 and 4G2 in pull-down precipitate. (*G*–*I*) Quantification of gRNA (*G*), sfRNA (*H*), and sfRNA:gRNA ratios (*I*) in A549 cells at 2 hpi, after XRN1 knockdown. (*J*) Western blots of phosphorylated IRF3, IRF3, and LAMP-1 at 6 hpi with MOIs of 1 and 10 of mock, PR1, and PR1NS53UTR and cotreatment of NITD008. (*K*) Level of infection with MOIs of 1 and 10 of mock, PR1, and PR1NS53UTR quantified by immunofluorescence assay. Data are presented as mean ± SD. *P < 0.05; **P < 0.01; ***P < 0.001; ***P < 0.0001, unpaired *t* test. ns, not significant.

We had previously suggested that a higher sfRNA-to-gRNA ratio provides a “one-two punch” for DENV against host cell antiviral response—less gRNA would result in a lesser degree of RIG-I activation, while more sfRNA would inhibit any RIG-I signaling to a greater extent. However, de novo sfRNA synthesis occurs after gRNA replication. RIG-I signaling inhibition would conceptually be more effective if sfRNA were delivered to infected cells to inhibit RIG-I signaling before gRNA replication ensues. Packaging of sfRNA into infectious particles is plausible, as a recent study showed that the 3′ UTR serves as a signal for nucleocapsid assembly (23, 24). To explore this possibility, we measured gRNA and sfRNA in the culture supernatant of infected cells (Fig. 4*A* and *SI Appendix*, Fig. S4 *A*–*F*). Interestingly, PR2B, PR1NS5, and PR1NS53UTR showed higher sfRNA levels compared with gRNA levels in the culture supernatant of infected A549 cells (Fig. 4*A*) and primary monocytes (*SI Appendix*, Fig. S4*F*). We validated this result by Northern blot analysis (*SI Appendix*, Fig. S4*C*), using PR1NS53UTR as a representative. We also observed distinct bands above the sfRNA band, which could be indicative of defective interfering viral particles, as described previously (25).

We next explored whether sfRNA in the culture supernatant existed in solution or packaged within extracellular vesicles (EVs). EVs, in the form of either microvesicles (MVs) or exosomes (26), are products of cellular vesicles that are secreted from cells via exocytosis, which could contain both viral RNA and virions (26–28). Thus, we measured the levels of both gRNA and sfRNA in the EVs. The larger diameter of MVs compared with exosomes and virions (>100 nm vs. ∼50 to 100 nm) allows for separation of MVs from exosomes and virions by centrifugation (*SI Appendix*, Fig. S4*K*). Lower numbers of gRNA and sfRNA copies were observed in the pelleted fraction containing MVs compared with fractions with smaller vesicles (*SI Appendix*, Fig. S4 *L*–*N*), suggesting preferential packaging of gRNA and sfRNA in either virions or exosomes. Thus, we knocked down tetraspanins in A549 cells to reduce exosome formation. If sfRNA were packaged in exosomes alone, then knocking down tetraspanin expression would reduce sfRNA abundance relative to gRNA abundance in the supernatant. Silencing of CD9, CD63, CD81, and CD151 in A549 cells resulted in lower gRNA copies (*SI Appendix*, Fig. S4 *G* and *I*) and sfRNA copies (*SI Appendix*, Fig. S4 *H* and *J*), but without the expected reduction in sfRNA:gRNA ratio in the supernatant for both PR1 and PR2B (Fig. 4*B*). While this finding supports previously published data indicating that DENV gRNA can be contained inside exosomes (27, 28), the experimental outcome suggests that sfRNA is not packaged primarily in exosomes.

To determine whether sfRNA is packaged in virions or infectious particles containing DENV envelope (E) protein and are thus infectious, we used a pan-DENV monoclonal antibody, 4G2, to enrich for E-containing infectious particles in the culture supernatant. An overview of the experiment is shown in *SI Appendix*, Fig. S5*A*. In brief, optimized levels of magnetic bead-conjugated monoclonal antibody 4G2 that binds DENV E protein (*SI Appendix*, Fig. S5*B*) was incubated with the culture supernatant of PR1- and PR2B-infected cells. The precipitate was subjected to RNase treatment (*SI Appendix*, Fig. S5*C*) to remove unpackaged viral RNA before viral envelope was digested for viral RNA extraction. RT-qPCR showed that gRNA copies were detectable in all viruses except PR1NS5 (Fig. 4*C*). Interestingly, sfRNA copies were detected in PR1NS5, PR1NS53UTR, and PR2B but not in PR1 and PR13UTR (Fig. 4*D*). Correspondingly, sfRNA:gRNA ratios in PR1NS5, PR1NS53UTR, and PR2B were increased (Fig. 4*E*). These findings suggest that sfRNA could be packaged in infectious particles. However, the 4G2 pull-down precipitate also contained exosomes (CD63) (Fig. 4*F*). To delineate whether sfRNA is packaged exclusively in virions, we used CD63 antibodies to deplete exosomes from culture supernatants before subjecting them to 4G2 pull-down (*SI Appendix*, Fig. S5*A*). While exosomes could be removed from PR1 culture supernatant, they could not be fully depleted in PR2B culture supernatant (*SI Appendix*, Fig. S5*D*), and sfRNA:gRNA ratios remained higher in PR2B compared with PR1 (*SI Appendix*, Fig. S5 *E–G*). Taken together, these findings suggest that sfRNA may be packaged in DENV E protein-containing particles, including both exosomes and virions.

Regardless of whether sfRNA was packaged in exosomes, virions, or both, the presence of DENV E protein raises the possibility that sfRNA could be delivered to susceptible cells via infection. To explore this possibility, we infected A549 cells and measured sfRNA levels at 2 hpi, before detectable gRNA replication ensued (29). To further reduce the likelihood of de novo sfRNA synthesis in our experiment, we also silenced XRN1 in the inoculated cells. Reduced XRN1 expression did not affect the levels of either gRNA or sfRNA at 2 hpi (Fig. 4 *F* and *G*), suggesting that sfRNAs were in the inoculum and not de novo synthesized. Furthermore, consistent with our findings in the 4G2-enriched fraction of the culture supernatant, only PR2B sfRNA could be detected in infected cells (Fig. 4*G*). Correspondingly, a higher sfRNA:gRNA ratio was observed in PR2B-infected cells (Fig. 4*H*).

Finally, we tested whether the packaged sfRNA can indeed inhibit the type I IFN response directly on delivery to new susceptible cells. We infected A549 cells cotreated with NITD008 at a concentration of 27 μM to ensure inhibition of viral replication and de novo synthesis of sfRNA. We then assessed the levels of phosphorylated IRF3 at 6 hpi. Infection at an MOI of 1 resulted in few to no infected cells at 6 hpi; however, at an MOI of 10, where most of the cells were infected (Fig. 4*K*), we observed a reduced level of phosphorylated IRF3 in PR1NS53UTR compared with PR1 (Fig. 4*J*), indicating early inhibition of the type I IFN response. Taken together, our data hint at the tantalizing possibility that sfRNA can be packaged in DENV E protein-containing infectious particles and delivered into cells for direct inhibition of the type I IFN response but in a DENV strain-dependent manner.

## Discussion

Epidemiologic observations have long suggested that within DENVs exist strains with greater levels of epidemiologic fitness to spread epidemically in natural human-mosquito-human cycles (5–10, 30). The genetic determinants of such epidemiologic fitness have remained vague, however. The 1994 dengue epidemic in Puerto Rico, in which the emergence of a new clade of DENV2, PR2B, that displaced the endemic PR1 clade, provides a context in which to identify the genetic determinants of the epidemiologic fitness of DENV (12). Here we add to this body of knowledge and suggest that a reduced gRNA replication rate through the 5-aa substitution in the NS5 protein influences the relative abundance of sfRNA that enabled it to compete favorably with gRNA for packaging into infectious particles.

Our Bayesian molecular dating analysis revealed that PR2B 3′ UTR mutations were acquired well before the 1994 outbreak. Mutations in the coding region (NS1, NS3, and NS5) of PR2B were acquired after those in the 3′ UTR. These mutations also could have contributed to the emergence of the 1994 dengue outbreak. Indeed, we recently found that another amino acid mutation, T164S, this time in the NS1 protein, resulted in increased secretion of hexameric NS1 (31), which has been shown to be a mediator of vascular leakage (32). Consequently, this T164S mutation also could have contributed to more severe dengue and hence more healthcare-seeking patients. However, of the eight mutations that segregated PR2A from PR2B, five (62.5%) were in the NS5 gene. The largest multifunctional NS protein in the DENV genome, NS5 consists of an N-terminal methyltransferase domain and a C-terminal RdRp domain connected by a 10-aa-long linker (33, 34). NS5 RdRp enables de novo RNA genome synthesis, and the methyltransferase caps the newly synthesized genome. Thus, we focused our attention on the significance of these NS5 mutations in altering the epidemiologic fitness of DENV2. Indeed, previous studies have shown that mutations in NS5 compromise virus replication (35, 36).

Of the five PR2B NS5 mutations, four were located in the RdRp domain (K375R, R596K, R514K, and R891K) and one was located in the linker region (S269N). DENV NS5 RdRp binds the 5′ UTR stem-loop A for initiation of de novo synthesis of the viral genome (37–39). Introduction of PR2B NS5 mutations into the PR1 genomic backbone produced mutants with lower rates of gRNA replication. Since an arginine patch in the RdRp may play a role in facilitating viral RNA binding (20), the R-to-K substitutions (R596K, R514K, and R891K) in the RdRp domain of PR2B NS5 could have reduced the efficiency of gRNA binding to the polymerase, thereby reducing the gRNA replication rate. Reduced gRNA could be important to ensure that XRN1 levels are in excess of the uncapped gRNA substrate to generate more sfRNA relative to gRNA. Indeed, this interpretation is well supported by our experimental finding that inhibition of PR1 NS5 polymerase activity using the previously validated antiviral compound, NITD008, increased the sfRNA:gRNA ratio in PR1- and PR13UTR-infected cells.

We have previously suggested that the increased relative abundance of sfRNA compared with gRNA served as a “one-two punch.” We now show that that sfRNA could be delivered in infectious particles and serve as a form of “preemptive strike.” Delivery of sfRNA in infectious particles would inhibit RIG-I before de novo gRNA replication, an increased abundance of which would stimulate RIG-I. Histologically, DENV replication and encapsidation occur concurrently in vesicle packets (VPs) on membranes of the endoplasmic reticulum (ER) (40). Synthesized negatively charged viral RNA exits through VP pores and is transported into virus-budding sites in the ER for encapsidation by the positively charged DENV capsid protein (40). A recently reported study found that specific binding of NS2A to the last 285 nucleotides of the 3′ UTR facilitates transport of the viral RNA to the virion assembly site (23). As sfRNA formation follows gRNA replication and thus is also localized within the reorganized ER, excess sfRNA could conceivably compete with gRNA for packaging or even encapsidation into infectious particles or virions. To our knowledge, this finding has not been reported previously. The literature only describes how sfRNA is formed and characterizes its multifaceted role in immune evasion through viral RNA–host protein interactions (12, 14, 41–43). Our results also show that packaging of sfRNA into infectious particles is dependent on the strain of DENV, as sfRNA levels must far exceed gRNA levels. Serial passage and early harvest of DENVs in cell cultures may select against slower-replicating but sfRNA-packaging DENV strains. This possibility underscores the need to use low-passaged clinical isolates to examine the epidemiologic fitness of DENV.

Our data collectively propose a model to explain the Puerto Rico dengue outbreak in 1994. Mutations in the 3 ′UTR were acquired possibly as early as 1985, although the type I IFN repression phenotype could not be revealed until the mutations in the NS5 were acquired just before the 1994 outbreak. We speculate that if the NS5 mutations had come before the 3 ′UTR mutations, such a strain would not have survived; reducing the rate of gRNA replication without the ability to effectively suppress type I IFN expression likely would have resulted in a DENV strain that was unable to achieve sufficiently high viremia levels to infect blood-feeding *A. aegypti*. Instead, acquisition of the 3′ UTR mutations in the backbone of a more rapidly replicating virus allowed this genotype to be sustained until acquisition of the NS5 mutations that produced a sfRNA:gRNA ratio favorable for sfRNA packaging or encapsidation. Our findings suggest a role for positive epistasis between NS5 and the 3′ UTR to reveal a “preemptive strike” function of DENV sfRNA for increased epidemiologic fitness.

## Materials and Methods

### Cell Lines.

Hek293T (ATCC), A549 (ATCC), and Huh7 (Duke Cell Repository) cells were cultured in Dulbecco’s Modified Eagle Medium (DMEM) with 10% FBS. BHK (ATCC) and C6/36 (ATCC) cells were cultured in RPMI 1640 (Gibco) with 10% FBS.

### Isolation of Primary Monocytes.

Peripheral blood mononuclear cell (PBMC)-derived primary monocytes were isolated from flavivirus-naïve healthy donors according to a protocol approved by the National University of Singapore’s Institutional Review Board (reference no. B-15-227) as described previously (44). Written consent was obtained from all donors, and all samples were deidentified before use in experiments.

### Synthesis and Mutagenesis of Infectious Clones.

DENV2 infectious clones were constructed using the Gibson assembly method (45, 46). In brief, viral RNA was extracted from strain PR1940 (representing the Puerto Rico endemic clade PR1) and strain PR8541 (representing the Puerto Rico epidemic clade PR2B) using the QIAamp Viral RNA Mini Kit (Qiagen) according to the manufacturer’s instructions. cDNA was synthesized using the SuperScript III First-Strand Synthesis kit (Invitrogen), and 2 µL of this cDNA was used to synthesize six DNA fragments that amount to the complete DENV genome using Q5 Hotstart 2× Master Mix (New England BioLabs). PCR products were then gel-extracted and purified using the QIAquick Gel Extraction Kit (Qiagen) according to the manufacturer’s instructions, and TA cloning was performed using pGEM-T Easy Vector (Promega). The QuikChange Multi Site-Directed Mutagenesis Kit (Agilent) was used to incorporate the single site mutations in NS5. Equimolar (0.1 pmol) of pUC19 vector containing the human cytomegalovirus (CMV) promoter and simian virus 40 polyadenylation signal (SV40-pA) and each fragment were assembled with the NEBuilder HiFi Assembly Kit (New England BioLabs) at 50 °C for 1 h.

The assembled products were transfected into Hek293T cells using Lipofectamine 2000 (Invitrogen) and Opti-MEM (Gibco). In brief, 5 µL of assembled product was used for transfection, and supernatant was collected 48 h later. Infectious clones were propagated in C6/36 cells supplemented with 3% FBS. Infected cells were observed daily for syncytia (∼3 to 7 d), harvested, and stored at −80 °C until further use. Viral titers were determined by plaque assay. Infectious clones used in subsequent experiments were passaged one to three times in C6/36 cells. Synthesized infectious clones were confirmed by Sanger sequencing as described previously (47) and analyzed using Geneious 9.0.5 (48).

### Phylogenetic Analysis.

Whole-genome sequences of the Puerto Rico DENV2 were retrieved from the NIAID ViPR (18). The dataset was imported into Geneious 9.0.5 (48), and multiple sequence alignment was performed using the MAFFT plugin (49). After alignment, the dataset was scanned to identify any incomplete sequences or sequencing errors that may have resulted in gaps.

An ML tree was generated according to the GTR nucleotide substitution model using RAxML (16). PAML (17) was used in conjunction with RAxML to map the synonymous and nonsynonymous amino acid mutations along the branches of the tree.

For the dating analysis, the ML tree was screened using TempEst (50), and any outlier strains were removed before analysis. A molecular clock was estimated using the uncorrelated relaxed clock model in a Bayesian Markov chain Monte Carlo framework in BEAST 1.8.3. The analysis was performed using the GTR nucleotide substitution model with a gamma distribution of among-site rate variation (GTR+G) and a Gaussian Markov random field Bayesian Skyride coalescent model. The dataset was analyzed for 200 million generations, with sampling every 10,000 runs. After removal of an appropriate burn-in of 10% of the runs, an MCC tree was inferred and visualized with FigTree 1.4.2.

### Virus Quantification.

A plaque assay was performed to quantify viral titers as described previously (44) using maintenance RPMI 1640 on BHK21 cells. Viral titers were calculated as pfu/μL.

### Virus Infection.

Assessment of virus replication was performed in Huh7 cells, A549 cells, or PBMC-derived monocytes. Cells were infected with the virus at an MOI of 1 unless stated otherwise and the incubated for 1 h at 37 °C. After incubation, the inoculum was removed and replaced with DMEM with 3% FBS and 1% penicillin-streptomycin. Cell supernatant and cell lysates were harvested at different time points. RNA extraction of cell lysates and supernatant were done using the RNeasy Mini Kit and QIAamp Viral RNA Mini Kit, respectively (Qiagen) according to the manufacturer’s instructions. Next, cDNA synthesis was performed using the qScript cDNA Synthesis Kit (Quantas Biosciences). Viral replication kinetics were measured by RT-qPCR using the SYBR Green Supermix Kit (Roche). All reactions were run on a Roche LightCycler 480, and data analysis was performed with LightCycler 480 software. The amount of viral RNA present in cell lysates was calculated as the gRNA:GAPDH ratio. The sfRNA:gRNA ratio was quantified according to a previously established method (41). The following primers were used to detect gRNA and sfRNA: gRNA forward, 5′-CCA​TGA​AGA​GAT​TCA​GAA-3′; sfRNA forward, 5′-GGA​CGT​TAA​AAG​AAG​TCA-3′; gRNA/sfRNA reverse, 5′-GCT​GCG​ATT​TGT​AAG​GG-3′.

### Northern Blot Analysis.

Huh7 cells were infected as described above. Cell lysate was harvested at 24 hpi for viral RNA extraction using TRIzol (Life Technologies). Samples were run on a 1% formaldehyde denaturing gel and then transferred to a positively charged nylon membrane (Bio-Rad) overnight. Hybridization with α^32^P dUTP-labeled RNA probes was performed overnight using ULTRAhyb Ultrasensitive Hybridization buffer (Life Technologies) according to the manufacturer’s instructions. After hybridization, membrane was exposed on a phosphoscreen, and signals were quantified using a Typhoon phosphoimager (GE Healthcare Lifesciences). The primer pair 5′-ATA​AAG​CTT​TAA​TAC​GAC​TCA​CTA​TAG​GGA​GAA​CCT​GTT​GAT​TCA​ACA​GCA​C-3′ and 5′-AGC CCC GTC CAA GGA CGT TAA AAG AAG TC-3′ was used to synthesize a fragment amplifying the 3′ UTR of the DENV2 genome from the whole DENV2 genome cDNA. This fragment was subsequently used for in vitro transcription for synthesis of the α^32^P dUTP-labeled RNA probe.

### Immunofluorescence Assay.

Huh-7 cells were infected at an MOI of 10 of virus in serum-free DMEM. At 24 hpi, infected cells were fixed with ice-cold methanol for 15 min at −20 °C. Following fixation, cells were washed twice with PBS and then being stored at 4 °C. Nonspecific binding was blocked by 30 min of incubation with 1% [wt/vol] BSA/PBS before incubation of cells with primary antibodies against NS5 (5R3, 30 nM), followed by incubation with secondary antibodies coupled to Alexa Fluor 488 or Alexa Fluor 594 (Invitrogen). Cell nuclei were visualized using DAPI (Sigma-Aldrich, 1:10,000). Coverslips were mounted onto glass slides using ProLong Gold antifade reagent (Invitrogen).

### Western Blot Analysis.

Cells were lysed in Nonidet P-40 buffer. For denaturing, 1× Laemmli buffer (Bio-Rad) with 2-mercaptoethanol (Sigma-Aldrich) was added to samples, followed by boiling for 10 to 15 min. For the nondenaturing condition, 1× Laemmli buffer (Bio-Rad) was added to samples without boiling. Samples were subsequently run on polyacrylamide gel for 90 min at 90 V. On transfer, membranes were blocked with 1× PBS containing either 5% nonfat dry milk or 5% BSA. Primary antibodies were diluted in blocking buffer. The denaturing condition was applied for XRN1 (Bethyl; 1:2,500), CD63 (Abcam; 1:5,000), phosphorylated IRF3 (Cell Signaling Technology; 1:1,000), IRF3 (Cell Signaling Technology; 1:1,000), β-actin (1:5,000), and LAMP-1 (1:5,000), and the nondenaturing condition was applied for CD9 (Abcam; 1:500), CD81 (Abcam; 1:250), and CD151 (Thermo Fisher Scientific; 1:1,000). Secondary antibodies (anti-rabbit, 1:5,000, and anti-mouse, 1:5,000) were diluted in blocking buffer. Bands were visualized using ECL (Amersham) for chemiluminescence development.

### Knockdown Assay.

siRNA transfections were done using Lipofectamine RNAiMAX (Life Technologies) according to the manufacturer’s instructions on A549 cells for 72 h. Knockdown efficiencies were evaluated by Western blot analysis.

### NITD008 Infection Assay.

A549 cells were infected with virus at an MOI of 1 and cotreated with an ascending concentration of NITD008 from 1 μM to 3 μM, 9 μM, and 27 μM for 1 h at 37 °C. After incubation, the medium was changed to DMEM with 3% FBS and 1% penicillin-streptomycin containing the aforementioned concentrations of NITD008. Cell lysates were harvested 24 at hpi for RT-qPCR quantification of viral gRNA and sfRNA as described above.

### Virus Pull-Down Assay.

A549 cells were infected with infectious clones at an MOI of 1 and incubated for 1 h at 37 °C, with shaking every 15 min. After incubation, the medium was changed to DMEM with 3% FBS and 1% penicillin-streptomycin. Cell supernatant was harvested at 24 hpi and subjected to pull-down with Dynabeads (Invitrogen) conjugated with 4G2 antibody or CD63 antibody (Genentech) according to the manufacturer’s instructions. For CD63 depletion, culture supernatants were subjected to CD63 pull-down before pull-down with 4G2. The pull-down fraction was subsequently treated with RNAse, and viral RNA extraction was performed using the Qiagen QIAamp Viral RNA Mini Kit. Following RNA extraction, cDNA synthesis and RT-qPCR analysis were conducted as described above.

### Statistical Analysis.

All results are presented as mean ± SD of three independent biological replicates. Data analysis was done with GraphPad Prism 8 using the unpaired Student *t* test or one-way ANOVA. Statistical significance was achieved at *P* < 0.05. In the figures, *P < 0.05; **P < 0.01; ***P < 0.001; ****P < 0.0001; and ns represents nonsignificance (*P* > 0.05).

### Data Availability Statement.

All data generated in this study are included in the paper and *SI Appendix*.

## Supplementary Material

Supplementary File
