# Supplementary material for: Positive epistasis between viral polymerase and the 3′ untranslated region of its genome reveals the epidemiologic fitness of dengue virus
Source: Proc Natl Acad Sci U S A. 2020 May 4;117(20):11038–47. doi: 10.1073/pnas.1919287117 (PMC7245076; doi:10.1073/pnas.1919287117)
Supplement: Supplementary File [file pnas.1919287117.sapp.pdf]

PNAS
www.pnas.org

Supplementary Information for

Positive epistasis between viral polymerase and 3' untranslated region of its genome reveals epidemiological fitness of dengue virus

Ayesa Syenina[1,2], Dhanasekaran Vijaykrishna[3], Esther Shuyi Gan[1], Hwee Cheng Tan[1], Milly Choy[1], Tanamas Siriphanitchakorn[1], Colin Cheng[1], Subhash Vasudevan[1], Eng Eong Ooi[1,2,4,5*]

[1]Program in Emerging Infectious Diseases, Duke-NUS Medical School, 169857, Singapore
[2]Saw Swee Hock School of Public Health, National University of Singapore, 117549, Singapore
[3]Department of Microbiology, Biomedicine Discovery Institute, Monash University, Clayton VIC 3800, Australia
[4]Department of Microbiology and Immunology, Yong Loo Lin School of Medicine, National University of Singapore, 119228, Singapore
[5]SingHealth Duke-NUS Global Health Institute, 169857, Singapore

* Corresponding author: Eng Eong Ooi
Email: engeong.ooi@duke-nus.edu.sg

This PDF file includes:

Figures S1 to S5

www.pnas.org/cgi/doi/10.1073/pnas.1919287117

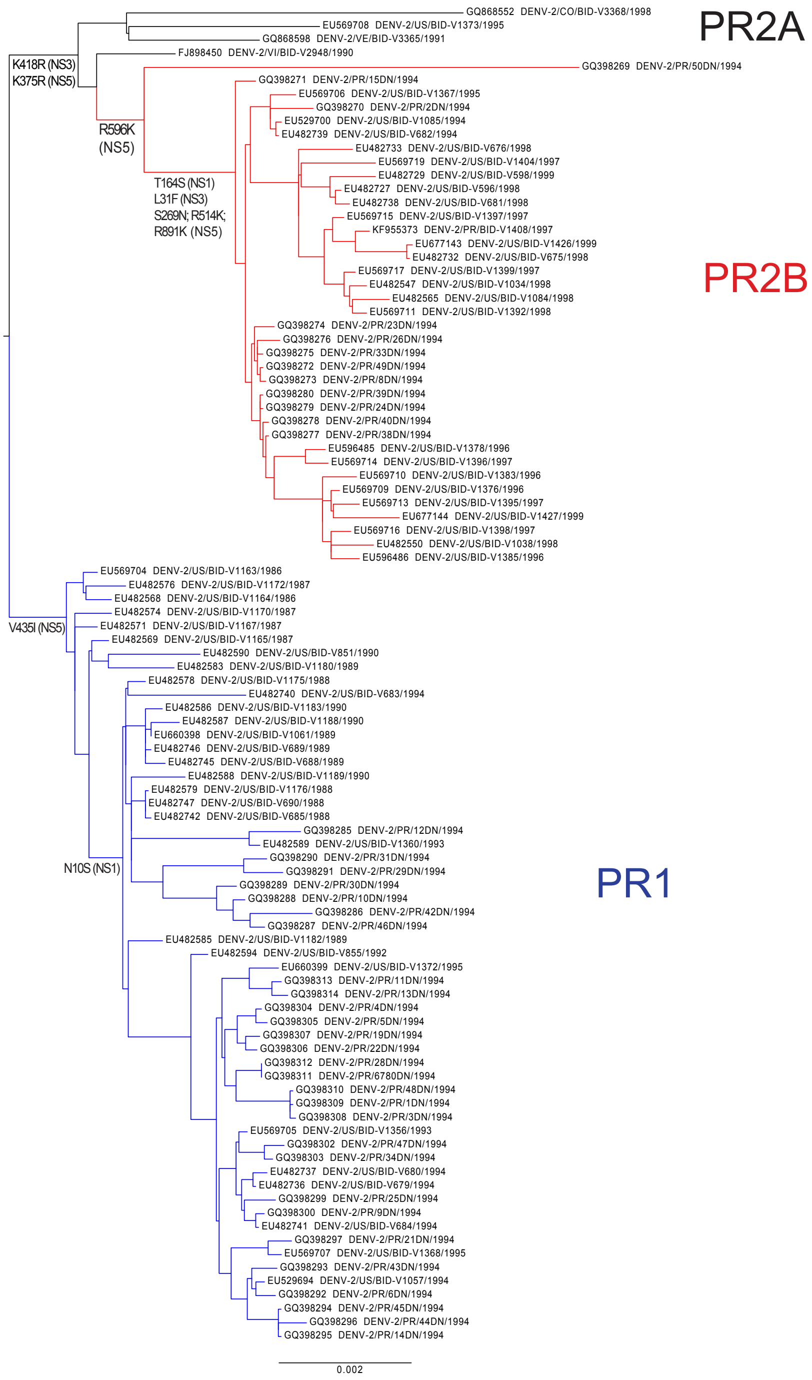

Supplementary Figure 1. Ancestral state reconstruction of Puerto Rico DENV2 viruses. Maximum likelihood (ML) phylogeny of the complete codon region of PR DENV2 viruses showing non-synonymous ancestral substitutions that segregate PR1 and PR2B. Two of these substitutions were in the NS1 (N10S and T164S), two in NS3 (L31F and K418R) and six in NS5 (S269N, K375R, V435I, R514K, R5967K, andR891K).

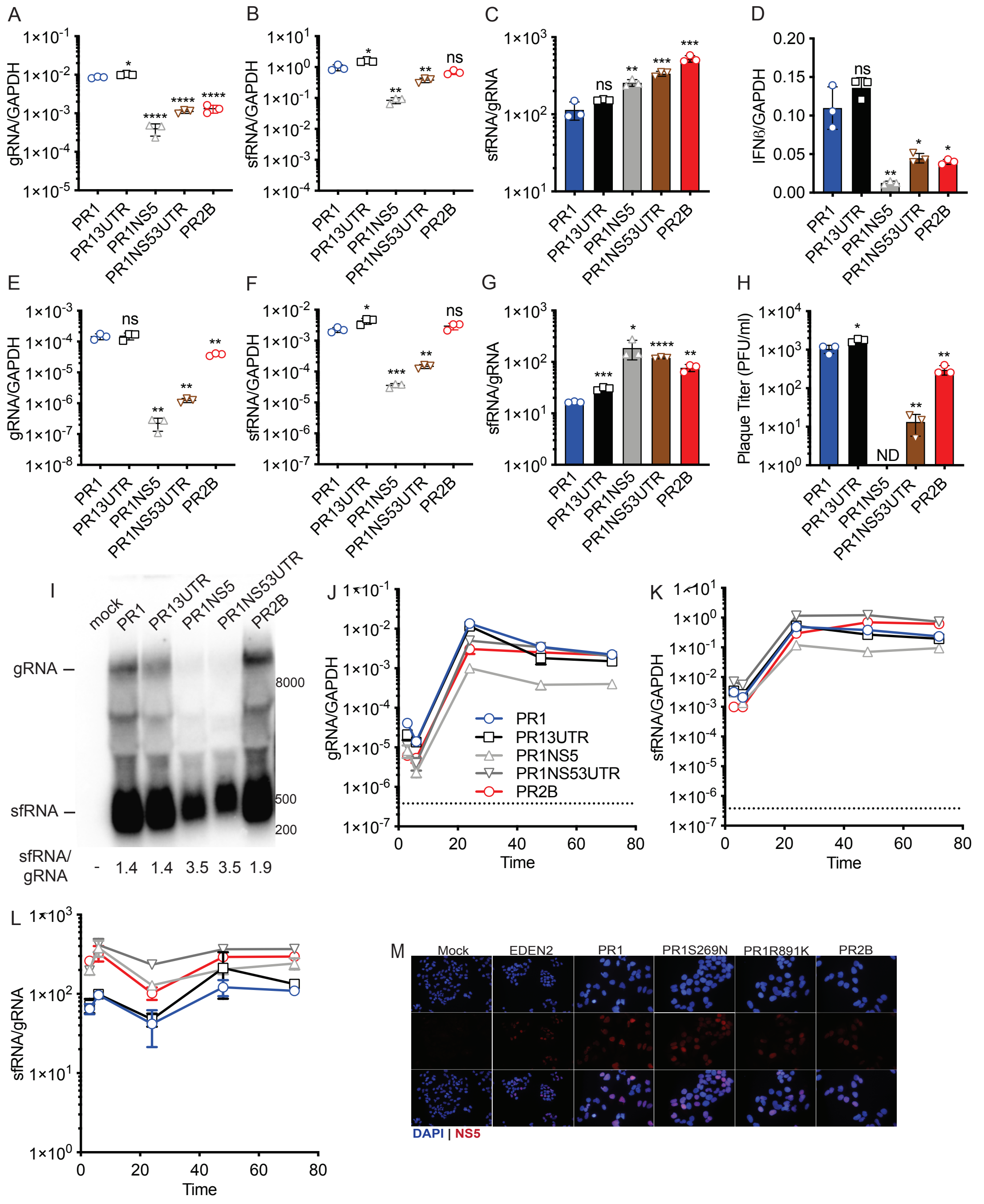

Supplementary Figure 2. NS5 mutations results in increased sfRNA:gRNA ratios. (A) Quantification of gRNA levels, (B) sfRNA levels, (C) sfRNA:gRNA ratios, and (D) mRNA expression of IFNβ in A549 cells, 24 hpi using qPCR. (E) Quantification of gRNA levels, (F) sfRNA levels, and (G) sfRNA:gRNA ratios in Huh7 cells, 24 hpi using qPCR; (H) PFU determined from supernatant of infected cells. (I) Northern blot analysis of gRNA and sfRNA produced in Huh7 cells; sfRNA:gRNA ratios from blot are shown. (J) Quantification of gRNA levels, (K) sfRNA levels, and (L) sfRNA:gRNA ratios from 3 hpi – 72 hpi using qPCR. (M) Immunofluorescence imaging of Huh7 cells stained for nucleus (DAPI in blue) and DENV2 NS5 in red. Data represented as mean ± s.d. *p<0.05, **p<0.01, ***p<0.001, and ***p<0.0001 (unpaired t-test); ns represents non-significance.

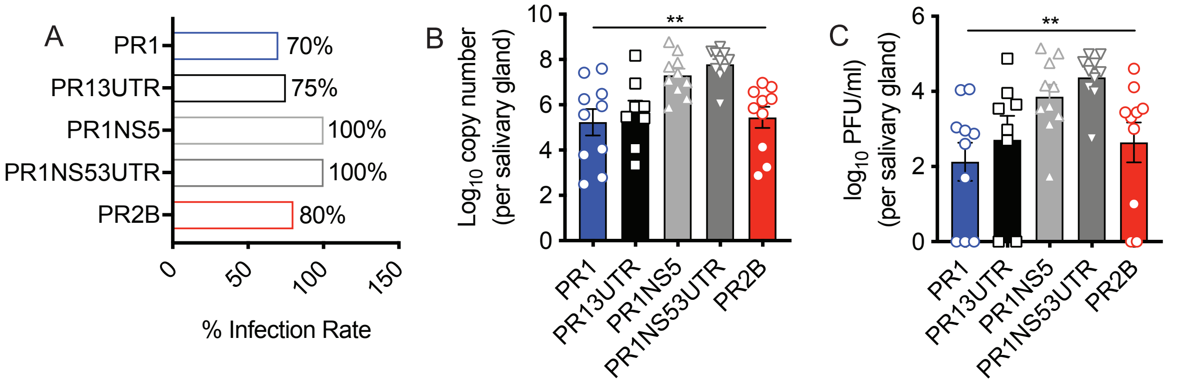

Supplementary Figure 3. Infection of viruses in *Aedes aegypti*. (A) Quantification of infection rate, calculated as percentage of infected mosquitoes with detectable plaques, (B) gRNA copy number per salivary gland, and (C) pfu per salivary gland from infected *Aedes aegypti* mosquitoes (n=8-10), 14 dpi using qPCR. Data represented as mean ± s.d. **p<0.01 (one-way ANOVA).

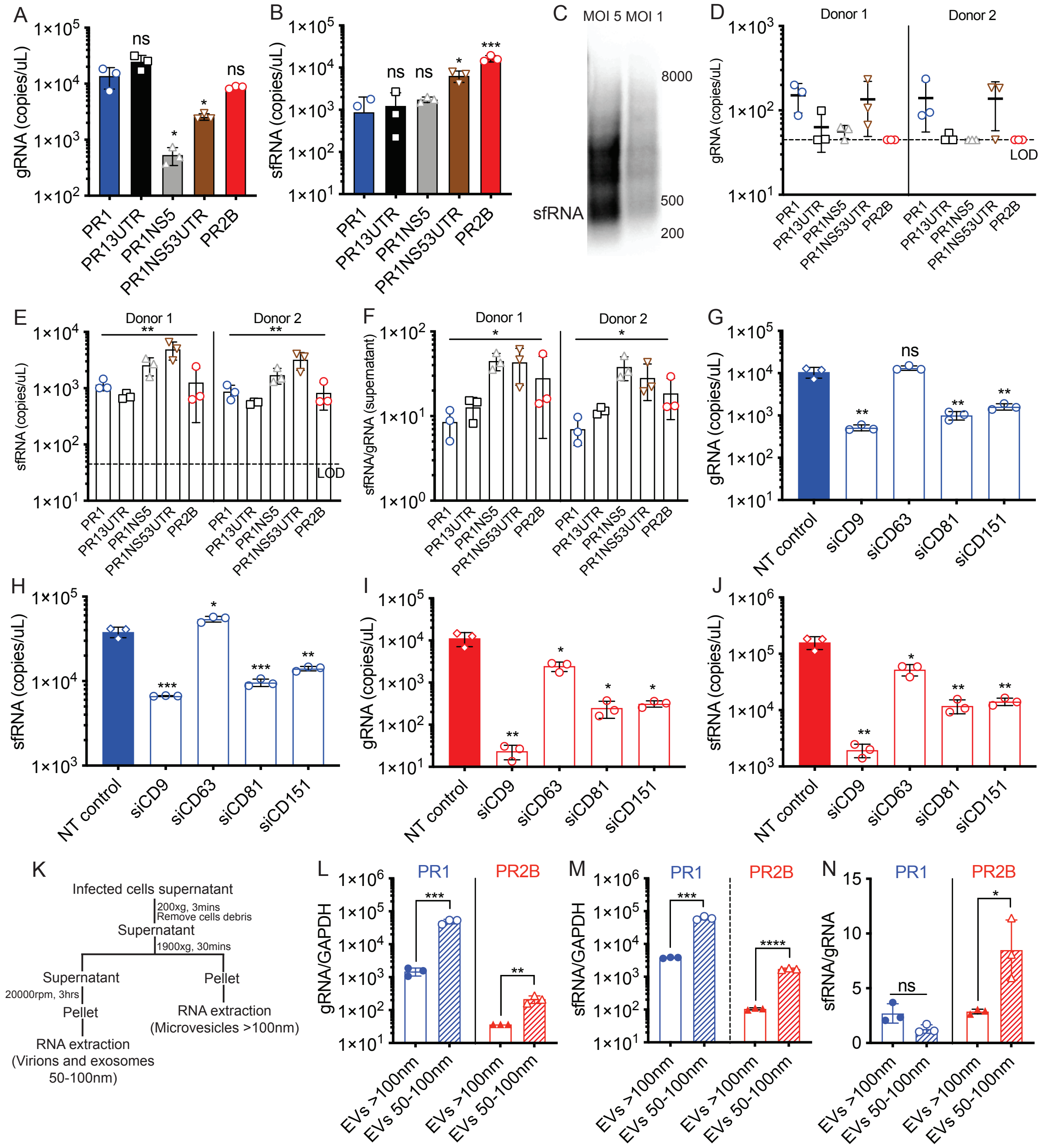

Supplementary Figure 4. PR2B sfRNA is detected in supernatant of infected cells. (A) Quantification of gRNA copy numbers and (B) sfRNA copy numbers in supernatant of A549 cells, 24 hpi using qPCR. LOD denotes limit of detection. (C) Detection of sfRNA in infected culture supernatant by northern blot. (D)Quantification of gRNA copy numbers, (E) sfRNA copy numbers, and (F) sfRNA:gRNA ratios in supernatant of primary monocytes, 72 hpi using qPCR. (G-J) Quantification of (G,I) gRNA copy numbers and (H,J) sfRNA copy numbers in supernatant of A549 cells upon knock-down of CD9, CD63, CD81, and CD151. NTC was used as a reference point for assessment of significance. (K) Schematic of centrifugation separation of microvesicles. (L) Quantification of gRNA levels, (M) sfRNA levels and (N) sfRNA:gRNA ratios in fraction containing microvesicles (>100nm in size) and fraction containing virions and exosomes (50-100nm in size). Data represented as mean ± s.d. *p<0.05, **p<0.01, ***p<0.001, and ***p<0.0001 (unpaired t-test); ns represents non-significance.

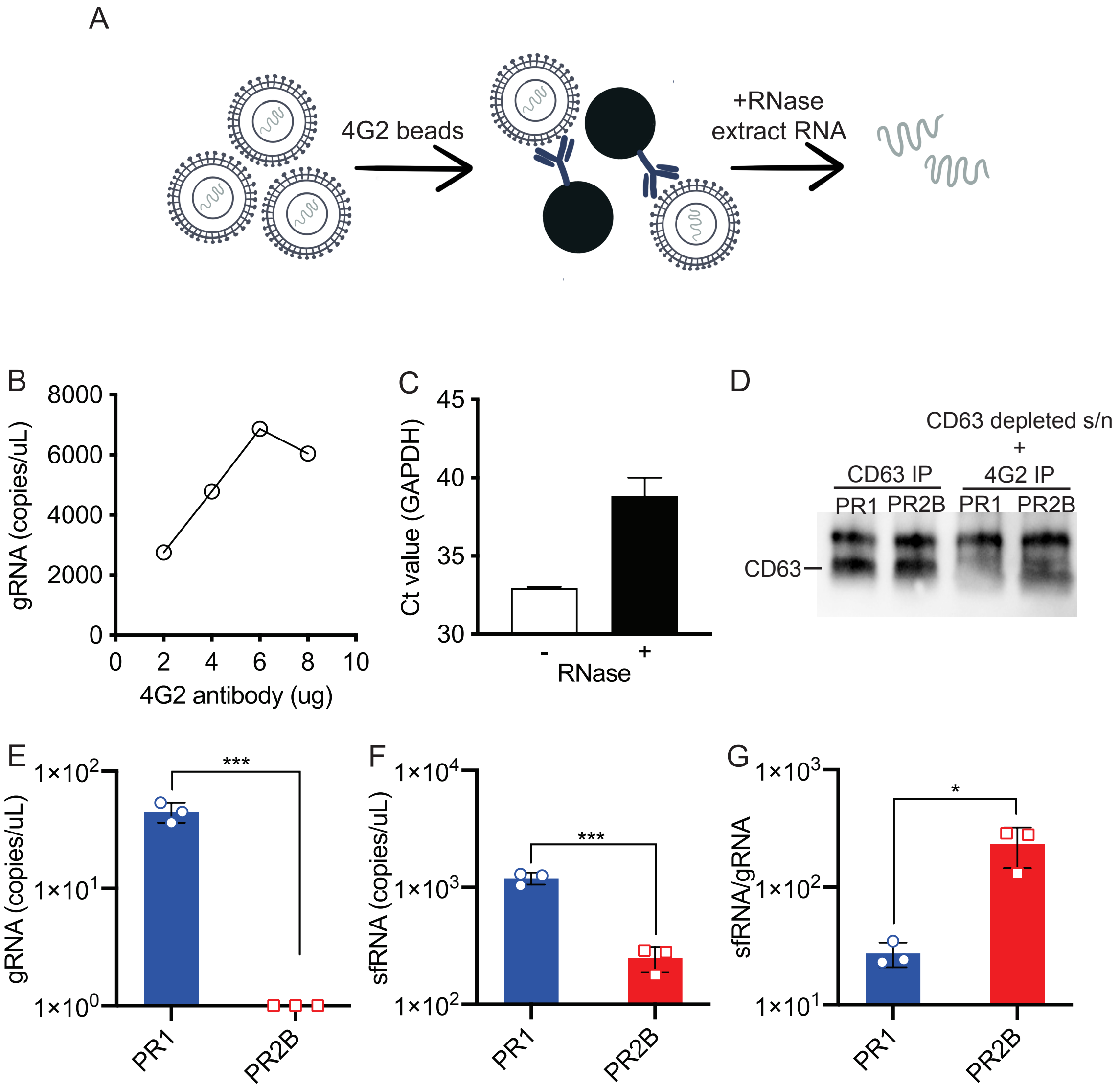

Supplementary Figure 5. PR2B sfRNA is encapsidated inside infectious particles. (A) Schematic of virion pulldown assay: supernatant from infected cells were incubated with 4G2-conjugated magnetic beads, following elution, pull-down fraction was subjected to RNase treatment and subsequently RNA extraction. (B) Optimization of 4G2 antibody concentration for virions pulldown. (C) cDNA samples were spiked with RNA to test for RNase activity. (D) Western blot of CD63 in CD63 pull-down precipitate and 4G2 precipitate after CD63 depletion. (E) Quantification of gRNA copy numbers, (F) sfRNA copy numbers, and (G) sfRNA:gRNA ratios from 4G2 pull-down precipitate after CD63 depletion. Data represented as mean ± s.d. *$p<0.05$, **$p<0.01$, ***$p<0.001$, and ***$p<0.0001$ (unpaired t-test); ns represents non-significance.
